# Omega-9 Modifies Viscoelasticity and Augments Bone Strength and Architecture in a High-Fat Diet-Fed Murine Model

**DOI:** 10.3390/nu14153165

**Published:** 2022-07-31

**Authors:** Mahmoud Omer, Hessein Ali, Nina Orlovskaya, Amelia Ballesteros, Vee San Cheong, Kari Martyniak, Fei Wei, Boyce E. Collins, Sergey N. Yarmolenko, Jackson Asiatico, Michael Kinzel, Christopher Ngo, Jagannathan Sankar, Ashley Calder, Timothy Gilbertson, Teerin Meckmongkol, Ranajay Ghosh, Melanie Coathup

**Affiliations:** 1 Department of Mechanical and Aerospace Engineering, University of Central Florida, Orlando, FL 32816, USA; ashourh1@knights.ucf.edu (H.A.); nina.orlovskaya@ucf.edu (N.O.); jasiatico@knights.ucf.edu (J.A.); michael.kinzel@ucf.edu (M.K.); ranajay.ghosh@ucf.edu (R.G.); 2 Biionix Cluster, University of Central Florida, Orlando, FL 32827, USA; ameliaroseball@gmail.com (A.B.); kari.martyniak@ucf.edu (K.M.); fei.wei@ucf.edu (F.W.); cngo24@knights.ucf.edu (C.N.); teerin.meckmongkol@nemours.org (T.M.); melanie.coathup@ucf.edu (M.C.); 3 College of Medicine, University of Central Florida, Orlando, FL 32827, USA; ascalder@med.umich.edu (A.C.); timothy.gilbertson@ucf.edu (T.G.); 4 Department of Automatic Control and Systems Engineering, Insigneo Institute for In Silico Medicine, University of Sheffield, Sheffield S1 3JD, UK; v.cheong@sheffield.ac.uk; 5 Engineering Research Center for Revolutionizing Biomaterials, North Carolina A&T State University, Greensboro, NC 27411, USA; becollin@ncat.edu (B.E.C.); sergey@ncat.edu (S.N.Y.); sankar@ncat.edu (J.S.); 6 Department of General Surgery, Nemours Children’s Hospital, Orlando, FL 32827, USA

**Keywords:** osteoporosis, saturated fats, polyunsaturated fats, monounsaturated, bone strength, viscoelasticity

## Abstract

The influence of diet on the development of osteoporosis is significant and not fully understood. This study investigated the effect of diets of varying lipid profiles and ω-3, ω-6 and ω-9 composition on the structural and mechanical properties of bone. The hypothesis studied was that a diet high in saturated fat would induce osteoporosis and produce an overall increased detrimental bony response when compared with a diet high in unsaturated ω-6, or ω-9. Male C57BL/6J mice were fed either a control diet, 50:50 mix (saturated:unsaturated) high in ω-9 (HFD^50:50^), a diet high in saturated fat (HSF) or a polyunsaturated fat diet high in ω-6 (PUFA) over an 8-week duration. Tibiae were retrieved and evaluated using DMA, 3-point-bending, histomorphometry, and microCT. Mice fed a HSF diet displayed key features characteristic of osteoporosis. The loss tangent was significantly increased in the HFD^50:50^ diet group compared with control (*p* = 0.016) and PUFA-fed animals (*p* = 0.049). HFD^50:50^-fed mice presented with an increased viscous component, longer tibiae, increased loss modulus (*p* = 0.009), and ultimate stress, smaller microcracks (*p* < 0.001), and increased trabecular width (*p* = 0.002) compared with control animals. A diet high in ω-9 resulted in an overall superior bone response and further analysis of its role in bone health is warranted.

## 1. Introduction

The incidence of osteoporosis-related fractures in North America is increasing dramatically and forecast to grow from 1.66 million to 6.26 million by 2050 [1,2]. Hip insufficiency fractures are prevalent and the annual cost of treating a hip fracture is projected to exceed USD 130 billion by 2050 [3]. While this increase is partially attributable to an aging population [4], it has been suggested that the increase in fracture occurrence is disproportionately high [5]. Among other factors (i.e., genetics and hormones), nutrition, including dietary fat, may contribute to increased susceptibility to osteoporosis-related insufficiency fractures [6,7,8]. Notably, diet is more closely related to bone fracture in older adults, suggesting food choices matter more in the older population [9]. The ingestion of the unhealthy Western diet; a diet characterized by processed and convenience foods that are high in saturated fats, sugars and salt, together with lack of fresh vegetables and whole grains, is flourishing. This diet is considered a major driver for the growing prevalence of obesity [10]. This may be of high significance as fat and bone tissue are linked by many pathways and new insights suggest that dysfunction at the cellular level (e.g., increased marrow adiposity, leptin, and pro-inflammatory cytokine release, with reduced osteoblastogenesis and levels of, e.g., osteopontin (OPN)), stimulates bone resorption and reduces bone mineralization and strength. Together this suggests that obesity is an important risk factor for osteoporotic fragility fractures [6,11,12,13,14]. In addition to the quantity of fat in the diet, the type of dietary fat may also be an important determinant of bone health. While saturated fat is typically regarded as a promoter of bone resorption and inhibitor of bone formation [6,12,13,14], a diet high in polyunsaturated fats (PUFAs) has been reported to significantly increase bone mineral content (BMC), bone mineral density (BMD) [15,16], bone stiffness, peak load and bone strength [17,18,19]. Polyunsaturated fats are classified into omega-3 (ω-3) and omega-6 (ω-6) fatty acids (FAs). Diets enriched with ω-3 are considered beneficial to bone health and are associated with a lower risk of fracture [20,21]. However, the influence of ω-6 FAs on bone health remains inconclusive. For example, in aging patients, diets high in ω-6 have been associated with increased BMD [22,23]. However, fracture risk has been reported to either decrease [21,24], increase [25,26], or have no effect [27]. Omega-9 (ω-9) FAs are monounsaturated (MUFAs) and very few studies have investigated ω-9 and bone health. To this end, a clinical study by Trichopoulou et al. [28] showed the ingestion of a diet high in MUFA-increased BMD, and, similarly, Martínez-Ramírez and colleagues reported a reduced fracture risk in their patient cohort [25]. In animal studies, high dietary levels of MUFAs have been associated with an increased trabecular volume fraction and thickness [29], with increased circulating osteocalcin (OCN), OPN and BMD [30]. However, and in contrast, Mozaffari et al. [31] described a significant positive association between high MUFA intake and increased risk of hip fracture. Many of the mechanisms that explain the impact of a high-FA diet on bone are still unknown.

To our knowledge, there are few studies that have directly compared the effect of a high-fat diet of varying saturated, monounsaturated and polyunsaturated levels on bone area, architecture, and mechanical behavior. The aim of this study was to investigate these parameters and contrast the effects of, (i) a high saturated fat diet, (ii) a high polyunsaturated diet (high in ω-6), and, (iii) a 50:50 mix (saturated:unsaturated) high in ω-9, on weight-bearing tibial cortical and cancellous bone in mice over an 8-week period. The hypothesis proposed was that a high saturated fat diet would induce osteoporosis and produce an overall increased detrimental bony response when compared to a PUFA diet high in ω-6, or a MUFA diet high in ω-9, over an 8-week duration.

## 2. Materials and Methods

All procedures involving animals were approved by the Institutional Animal Care and Use Committee at the University of Central Florida (protocol 2020-79; approved most recently in July 2022) and were performed in accordance with the American Veterinary Medical Associated guidelines. Male 8-week-old C57BL/6J mice were purchased from the Jackson Laboratory (Bar Harbor, ME, USA) and allowed to acclimatize for 2 weeks prior to dietary intervention. Mice were maintained on a 12:12 h light–dark schedule and given ad libitum access to food and water. Mice were randomized into experimental groups (*n* = 5). Body weights were recorded at the beginning of the study and then weekly until the end of the study. Similarly, food (g) and water (mL) intake were quantified by calculating the amount given and the amount remaining through each week, and until the end of the study.

### 2.1. Animals

Following acclimation, mice were challenged with either a (1) regular control diet, (2) high saturated fat diet (HSF), (3) high polyunsaturated fat diet (PUFA) or, (4) high-fat diet composed of a 50:50 mix of saturated:unsaturated FAs (HFD^50:50^) (Table 1). Each of the high fat diets provided 60 kcal% energy from fat. Fat ingredients included soybean oil, lard, hydrogenated coconut oil, safflower oil and cocoa butter, each introducing ω-3, ω-6 PUFA, and ω-9 MUFAs as well as a range of saturated fatty acids including stearic acid, palmitic acid, lauric acid and myristic acid (a full description is presented in Table 2). The mean kcal fraction of each component of the diet with respect to the total kcal was calculated. The percentage of ω-3 and ω-6 and ω-9 in each fraction was then estimated (g) to determine the contribution of ω-3 and ω-6 and ω-9 within each diet. 

**Figure 1:**
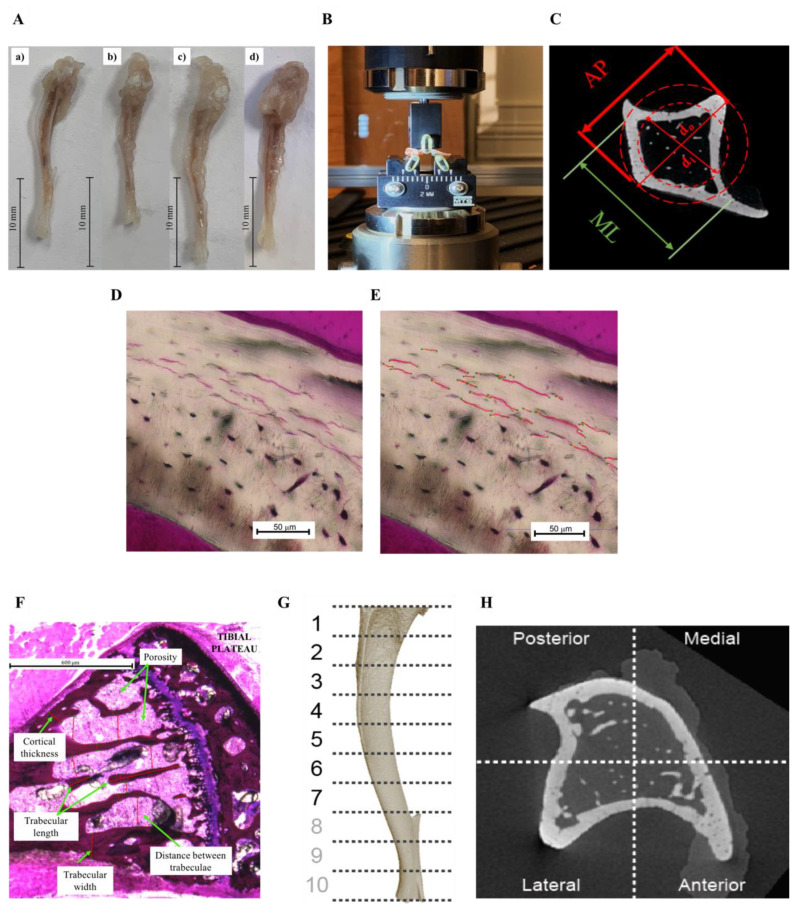
(**A**) Following euthanasia, tibiae were dissected for further analyses. Representative images of the tibia from (**a**) control, (**b**) HSF, (**c**) HFD^50:50^ and (**d**) PUFA groups. (**B**) Tibiae were loaded to failure under 3-point bending. (**C**) A transverse *μ*CT image of a tibia demonstrating the AP and ML directions, $d_{i}$ represents the inner diameter and $d_{o}$ the outer diameter. These values were used to calculate the biomechanical properties following 3-point testing. (**D**) A photomicrograph of cortical bone showing Basic Fuchsin stained microcracks (HSF group) under light microscopy. Image (**E**) shows measurements made (red lines) on longitudinally prepared sections using image analysis software (Keyence) and (**F**) the trabecular parameters measured within the proximal region of each tibia. (**G**) samples underwent *μCT* scanning and the tibia divided into 1 (proximal)–7 (tibio-fibular joint) regions along the tibial length and (**H**) in the AP and ML sectors.

Mice were euthanized 8 weeks post intervention and both the left and right tibiae were retrieved (Figure 1A). All right tibiae were plastic wrapped and stored frozen at −20 °C in preparation for microCT analysis and mechanical testing. All mechanical tests were carried out within 1 month of retrieval. All left tibiae were immediately immersed in 10% buffered formaldehyde before being processed for undecalcified histology. To evaluate the effect of each fat diet and the expected alterations in skeletal parameters, the following parameters measured (*n* = 5): (i) dynamic mechanical analysis (DMA) (storage loss (E’), loss modulus (E”) and loss tangent (δ)), (ii) 3-point bending until failure (ultimate stress (σu), fracture stress (σf), yield stress (σy) and elastic modulus (E)), (iv) tibial length (mm), (v) cross-sectional moment of inertia (m^4^), (iii) histological assessment (microcrack number and length (μm) in the cortical mid-shaft, bone viability). Cortical thickness, trabecular length, width, distance between trabecular (μm), %bone area and %porosity in the proximal tibia; measured in the region immediately beneath the growth plate and, (*n* = 1): (iv) micro-computed tomography (μCT) (BMD (mg.cm^−3^), BMC (mg) and BV/TV%).

### 2.2. Dynamic Mechanical Analysis

Dynamic loading of the bone was assessed using a Dynamic Materials Analyzer 242E (Artemis, Netzsch, Selb, Germany). Tibiae were thawed and immersed in phosphate-buffered saline (PBS) for at least 1 h prior to testing. The testing mode used was three-point bending, and experiments were performed isothermally. Each tibia was orientated such that the posterior aspect of the cortical midshaft was loaded at frequencies of 0.05, 0.1, 1 and 10 Hz, and under the elastic limit of bone. Stress was applied in a sinewave form with a constant stress amplitude of 0.25 MPa, where the maximum and minimum stresses were equal to 1 MPa and 0.5 MPa, respectively. Specimens were tested at room temperature for 60 min and the viscoelastic properties of each tibia (storage modulus E’, loss modulus E” and loss tangent (δ)) were obtained and results compared between groups.

### 2.3. Three-Point Bending 

Three-point-bending tests were performed using a universal testing machine (Criterion^®^ 43, MTS, Eden Prairie, MN, USA). Each tibia was loaded to failure at a displacement rate of 0.015 mm/s. The distance between the support bars was 6 mm, and each tibia was positioned perpendicular to the applied load with the anterior surface facing upwards (Figure 1B). A vertical force was applied to the mid-shaft using a 3 mm diameter loading roller (Figure 1B). The resulting load–displacement curves were then obtained. As the cross-sectional area of the tibia was non-uniform and similar to other studies [32,33], we assumed the cross-sectional area was circular and obtained the mechanical properties using the following equations.

(1) $$\sigma =\frac{F*L*c_{O}}{4*I}$$

(2) $$E=\frac{FL^{3}}{d*48*I}$$

where σ is the stress (Pa), F is the applied load (N), L = 0.006 is the span distance between the supports (m), $c_{o}$ is the outer radius of the tibia’s midshaft (m), which was measured using a calliper (Digital, Cole-Parmer, IL, USA), *E* is the elastic modulus (Pa), *d* is displacement (m) and *I* is the moment of inertia (m^4^), calculated as follows:

(3) $$I=\frac{\pi }{4\left(c_{o}^{4}-c_{i}^{4}\right)}$$

where $c_{i}$ is the inner radius of the tibial midshaft (m). The inner radius of the midshaft was measured from the μCT scans. Four different cross-sections were selected for one bone from each experimental group, and both inner and outer diameters were calculated from each cross-section as shown in (Figure 1C). The average value of inner-to-outer diameter from each cross-section were calculated to determine the inner radius. The yield point was determined using an offset of 0.015 mm parallel to the linear portion at the beginning of load–displacement plot [33]. The Post Yield Displacement (PYD) was obtained as the displacement from the yield point to the fracture point in the load–displacement plot. Yield stress, ultimate stress, fracture stress, elastic modulus, and cross-sectional moment of inertia were calculated. The mechanical strength parameters were adjusted for body size (ratio of body weight to tibial length) [34].

### 2.4. Histological Preparation 

Tibiae were dehydrated in serial dilutions of alcohol, and specimens were defatted and embedded in hard grade acrylic resin (LR White, Electron Microscopy Sciences, Hatfield, UK). Transverse thin sections (~60 µm) were prepared through the central midshaft of each tibia using a grinding and polishing technique (300CP and 400CS EXAKT system, Germany). The transverse sections were stained with Basic Fuchsin and the number and length of microcracks quantified using light microscopy and image analysis techniques (×20 objective lens, BZ-800E, Keyence, US) (Figure 1D,E). Additional sections were prepared longitudinally and through the center of the proximal and cancellous region of the tibia. Each longitudinal section was stained with Toluidine Blue and Paragon, which stained the soft tissue and bone, respectively. Using light microscopy and image analysis (×5 objective lens), (i) %bone area, (ii) trabecular thickness, (ii) trabecular length, (iii) distance between trabeculae, (iv) cortical thickness and (v) %porosity were measured (Figure 1F). Data were quantified and compared between each of the experimental groups. Scanning electron microscopy (SEM) (Jeol, Zeiss, Tescan) was used to assess bone viability and the presence of osteocytes within the lacunae. The surface of the embedded samples was polished and acid-etched with 9% phosphoric acid for 20 s, followed by washing in distilled water and 5% sodium hypochlorite. Samples were washed again in distilled water, dried overnight, and sputter coated prior to SEM viewing.

### 2.5. MicroCT

MicroCT scans were performed using a cone beam scanner (GE Phoenix Nanotom-M^TM^, Waygate Technologies). One tibia from each group was thawed and placed in 15 mL Eppendorf tubes and imaged at a 90 kV source voltage, 110 μA source current (mode 0) using a tungsten–diamond target with a 500 ms exposure time at 7–9 μm isotropic voxel resolution (depending on tibial size). Data were collected for 1080 projections over 360° (0.33° steps) with three averaged images per rotation position. The volume reconstructions were performed with Phoenix Datos software. Visualization and production of DICOM images was carried out using VG Studio Max (v 2.1) software. Samples were analyzed using Matlab 2018A (The MathWorks Inc., Natick, MA, USA), where a volume of interest (VOI) was selected immediately beneath the growth plate. Bone mineral density BMC, and bone volume fraction (BV/TV) were calculated in the anteroposterior and medio-lateral sectors in 1 (proximal)–7 (tibio-fibular joint) regions along one tibia in each group [35] (Figure 1G,H). In order to determine these parameters, density expressed as mg/cm^3^ of hydroxyapatite (HA) was determined using calibration phantoms of 0, 50, 200, 800 and 1200 mgHA/cm^3^. Three-dimensional models of the trabecular network within the proximal tibia were created using the 3D Slicer (v4.11.20210226; Brigham and Women’s Hospital and Massachusetts Institute of Technology). As only one mouse was μCT-analyzed for each group, the statistical analysis presented in the Supplementary Section was conducted by treating all of the longitudinal sections (1–7) as individual data points.

### 2.6. Statistical Analysis 

Analysis of the data was performed using SPSS software (v25; SPSS, Chicago, IL, USA). Data obtained were nonparametric and the Mann–Whitney U test was used for statistical comparison between experimental groups. The *p* values were corrected using Tukey’s HSD method, and *p* values < 0.05 were considered statistically significant. 

## 3. Results 

### 3.1. Food Intake and Bodyweight

**Figure 2:**
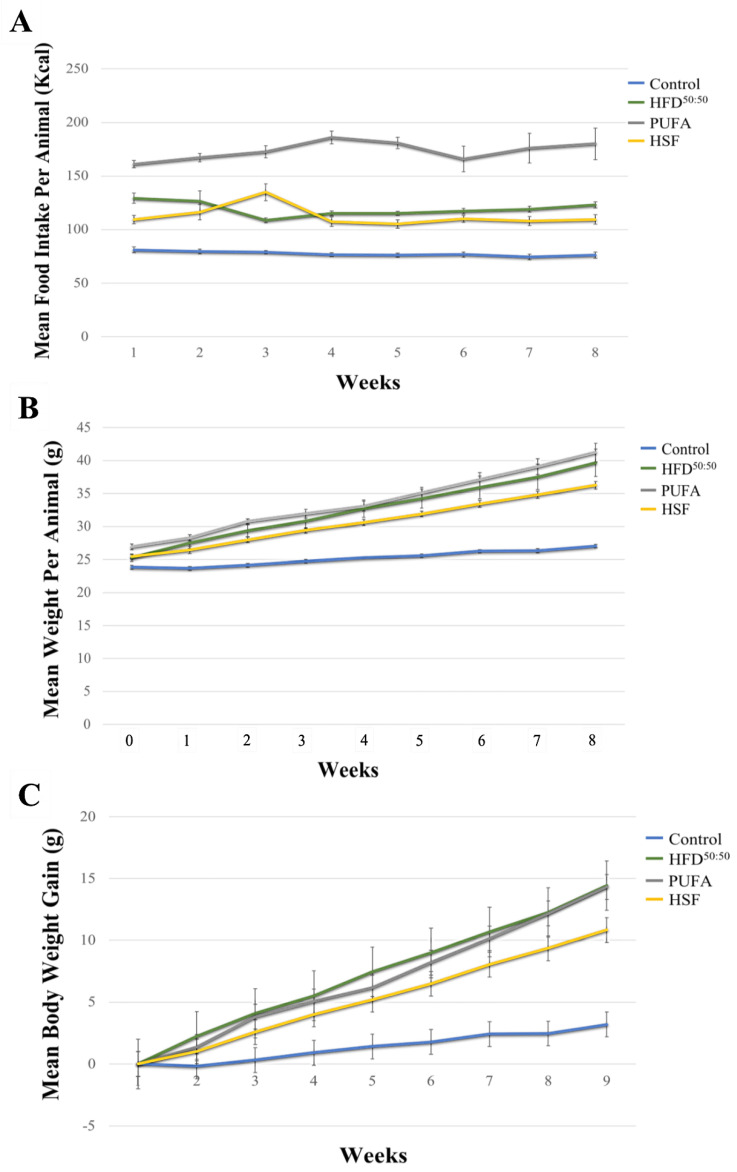
(**A**) Mean food intake (kcal), (**B**) mean weight per animal (g) and, (**C**) mean body weight gain (g), in each of the diet groups over the 8-week period. Data are expressed as mean ± SE. (*n* = 5 per group).

All animals remained healthy for the duration of the study. The mean food intake (kcal), body weight gain, and body weight per animal in each of the groups and over the 8-week study duration, is shown in Table 3 and (Figure 2A–C), respectively. Consumption of the HFD^50:50^, HSF, and especially, the PUFA diet, all led to significant weight gain when compared with control-fed animals (*p* < 0.05 in all groups). Animals in the PUFA group consumed significantly higher amounts of energy (kcal) when compared with animals in the control, HFD^50:50^ and HSF diet groups (*p* = 0.009 in all groups). No significant differences in water consumption were found. The *p* values obtained when food intake and body weight were statistically compared, and are presented in Supplementary Tables S1–S6.

### 3.2. Dynamic Mechanical Analysis 

**Figure 3:**
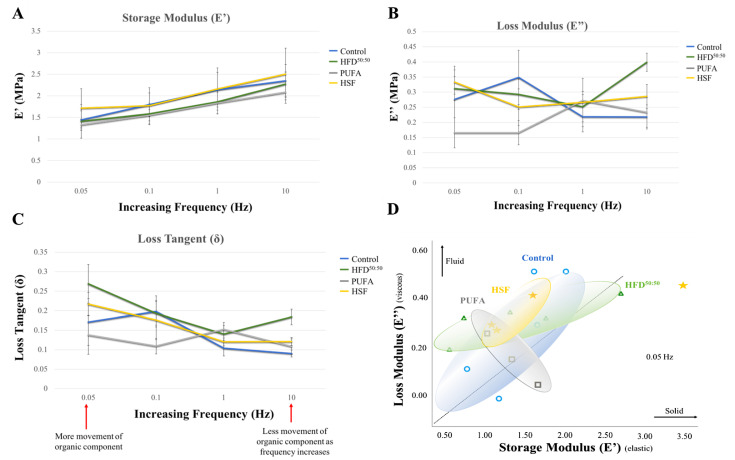
(**A**) Storage modulus, (**B**) loss modulus, (**C**) loss tangent in each of the diet groups. (**D**) A plot of loss modulus versus storage modulus in each of the groups. An increased viscous and reduced elastic component is demonstrated in the HSF and HFD^50:50^ groups when compared with control-fed mice. Data are expressed as mean ± SE. (*n* = 5 per group).

The results for storage modulus E’, loss modulus E” and loss tangent (δ) for control, HSF, HFD^50:50^ and PUFA groups at frequencies of 0.05, 0.1, 1 and 10 Hz are shown in (Figure 3A–C). Storage modulus increased in each group with increasing frequency. Results demonstrated a trend of a higher modulus in the HSF group, with lowest in the PUFA-fed animals. However, no significant differences were found. Changes in loss modulus were observed between groups. At the lowest stress level, a significantly lower loss modulus was measured in the PUFA group (0.17 ± 0.05 MPa) when compared with the HSF (0.33 ± 0.04 MPa, *p* = 0.027) and HFD^50:50^ (0.31 ± 0.04 MPa, *p* = 0.050) groups. At the highest frequency of 10 Hz, a significantly increased loss modulus was measured in the HFD^50:50^ group (0.40 ± 0.03 MPa) when compared with all other groups (control (0.22 ± 0.04 MPa), *p* = 0.009; HSF (0.29 ± 0.04 MPa), *p* = 0.047; and PUFA (0.23 ± 0.04 MPa), *p* = 0.049). At the highest stress levels, the mean values of the loss tangent were significantly increased in the HFD^50:50^ group (0.18 ± 0.02) when compared with the control group (0.09 ± 0.008; *p* = 0.016) and the PUFA-fed mice (0.11 ± 0.02; *p* = 0.049). Results demonstrated an increased viscous component in the tibia of mice fed with HFD^50:50^ (Figure 3D).

### 3.3. Three-Point-Bending

**Figure 4:**
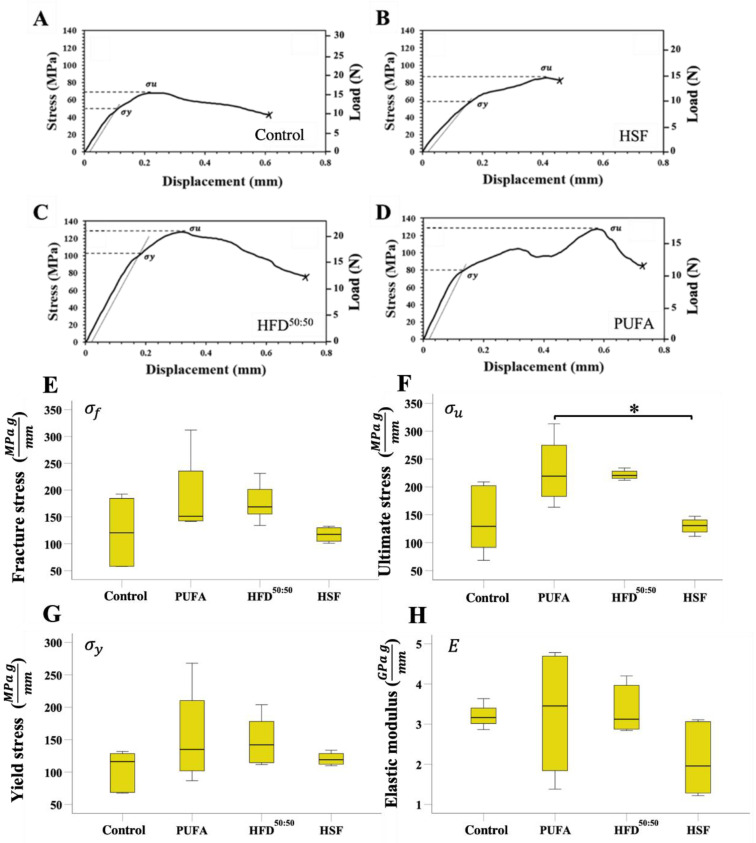
Data normalized to body weight and tibial length. (**A**–**D**) Representative stress-displacement deformation plots for tibiae in the control, HSF, HFD^50:50^ and PUFA groups. (**E**–**H**) The mechanical properties of strength $\sigma_{f}$, ultimate stress $\sigma_{u}$, yield stress $\sigma_{y}\;$ and elastic modulus *E* at the tibial mid-point, varied between the different diet groups (*n* = 5 per group) * *p* < 0.05.

Representative stress-displacement deformation plots for tibiae in the control, HSF, HFD^50:50^ and PUFA groups are shown in (Figure 4A). The tibiae exhibited varying deformation behavior when under the 3-point-bending test. “Pop-in” events were present, characterized as a decrease in the applied stress as the load increases, which is attributed to either the initiation of new microcrack formation or the propagation of existing microcracks within bone, until whole bone failure. The structural dependent properties of control, HSF, HFD and PUFA tibiae are shown in Table 4.

The mechanical properties of yield stress $\sigma_{y}$, ultimate stress $\sigma_{u}$, strength $\sigma_{f}$ and elastic modulus *E* at the tibial mid-point, varied between the different diet groups (Figure 4E–H). Results demonstrated that cortical strength at the mid-shaft was similar in the HSF- and control-fed animal groups. However, a trend was seen where mice fed a PUFA or HFD^50:50^ diet resulted in an increase in fracture stress, yield stress, and ultimate stress. Consumption of the PUFA diet over the 8-week period increased overall bone strength as fracture, ultimate, and yield stresses were all higher when compared with the other diet groups. Fracture stress was highest in the PUFA-fed mice (189.02 ± 82.35 MPa) and lowest in control-fed animals (122.71 ± 65.28 MPa). Ultimate stress was found to be similar in the control-fed (140.17 ± 63.50 MPa) and HSF-fed animals (140.16 ± 26.29 MPa). The highest ultimate stress measured in the PUFA group of animals (228.91 ± 63.68 MPa) was significantly increased when compared with the HSF group (*p* = 0.049). Tibiae in the HFD^50:50^ also displayed increased levels of ultimate stress (215.23 ± 16.75 MPa); increases which were not significant when compared to other groups. Similarly, yield stress levels had increased in the PUFA (156.11 ± 79.23 MPa), HFD^50:50^ (150.02 ± 40.29 MPa), and HSF (108.48 ± 28.20 MPa) groups when compared with the control-fed animals (102.52 ± 32.00 MPa). However, no significant differences were found. When the elastic modulus was evaluated, a trend was observed where the lowest modulus was measured in the HSF (2.12 ± 0.92 GPa) and PUFA groups (3.26 ± 1.69 GPa), when compared with the control (3.40 ± 1.64 GPa) and HFD^50:50^ animals (3.40 ± 0.63 GPA). No significant differences were found. Fracture, ultimate, yield stresses and elastic modulus data, unadjusted to body weight and tibial length, are presented in Supplementary Tables S7–S11 and Figure S1.

### 3.4. SEM and Histological Analyses

**Figure 5:**
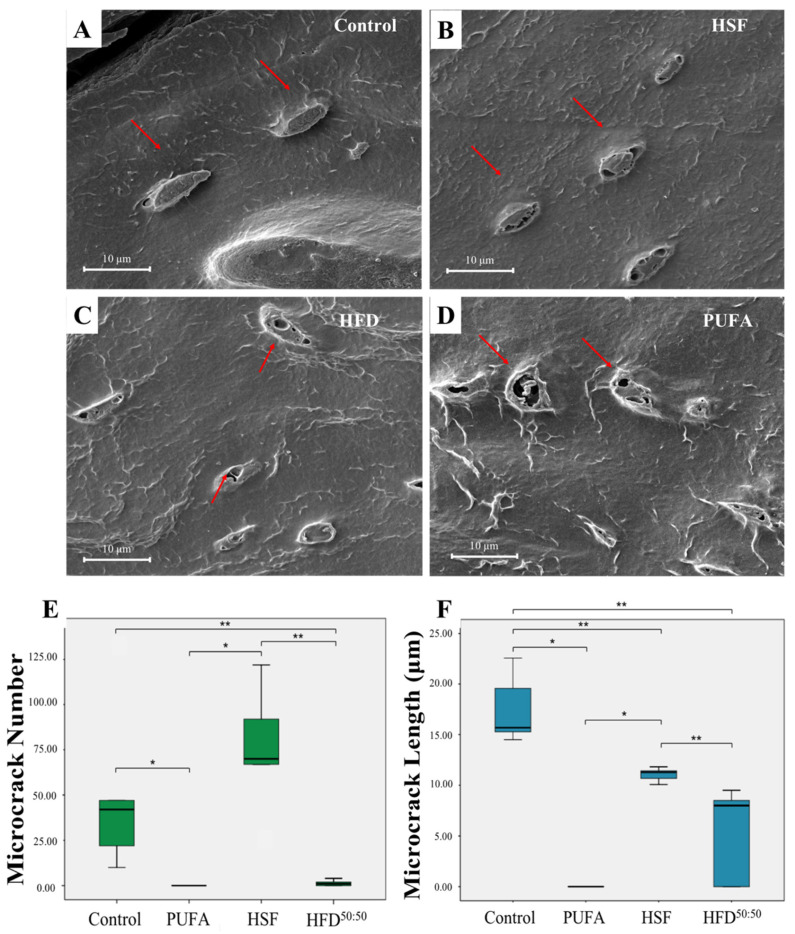
(**A**–**D**) Scanning electron micrographs showing osteocytes within their lacunae (red arrows) indicating viable bone. Scale bar = 10 μm. (**E**,**F**) microcrack number and length in each of the groups (*n* = 5 per group). * *p* < 0.05, ** *p* < 0.01.

When viewed using SEM, osteocytes were identified within lacunae in all groups indicating no qualitative differences in bone viability over the 8-week period (Figure 5A–D). Histological assessment of microcrack formation showed significantly increased microcrack numbers in the HSF group (94.6 ± 23.97) when compared with the HFD^50:50^ (1.40 ± 1.49, *p* < 0.001), PUFA (no cracks observed, *p* < 0.001), and control (50.6 ± 42.84, *p* < 0.001) groups (Figure 5E). The longest cracks were measured in the control diet group (17.51 ± 3.44) and smallest in the HFD^50:50^ group (5.20 ± 4.17). The length of microcracks was significantly reduced in the HFD^50:50^ diet and HSF groups when compared with control- and PUFA-fed animals (HFD^50:50^, *p* = 0.011; HSF, *p* = 0.037; PUFA, *p* = 0.009) (Figure 5F). 

**Figure 6:**
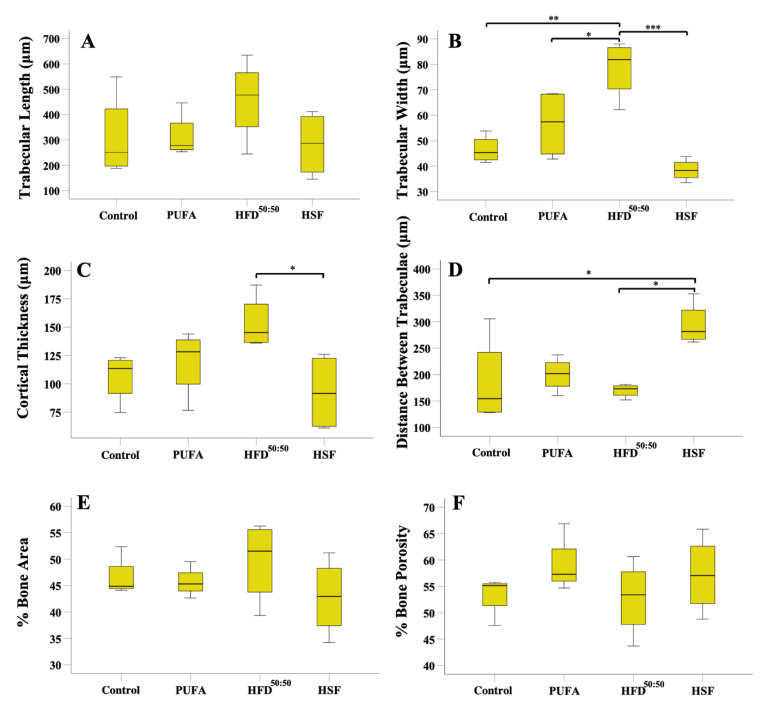
Histological analyses of bone in the cancellous region immediately below the growth plate. (**A**) trabecular length, (**B**) trabecular width, (**C**) cortical thickness, (**D**) distance between trabeculae, (**E**) %bone area and (**F**) %porosity in each of the groups (*n* = 5 per group). * *p* < 0.05, ** *p* < 0.01, *** *p* < 0.001.

**Figure 7:**
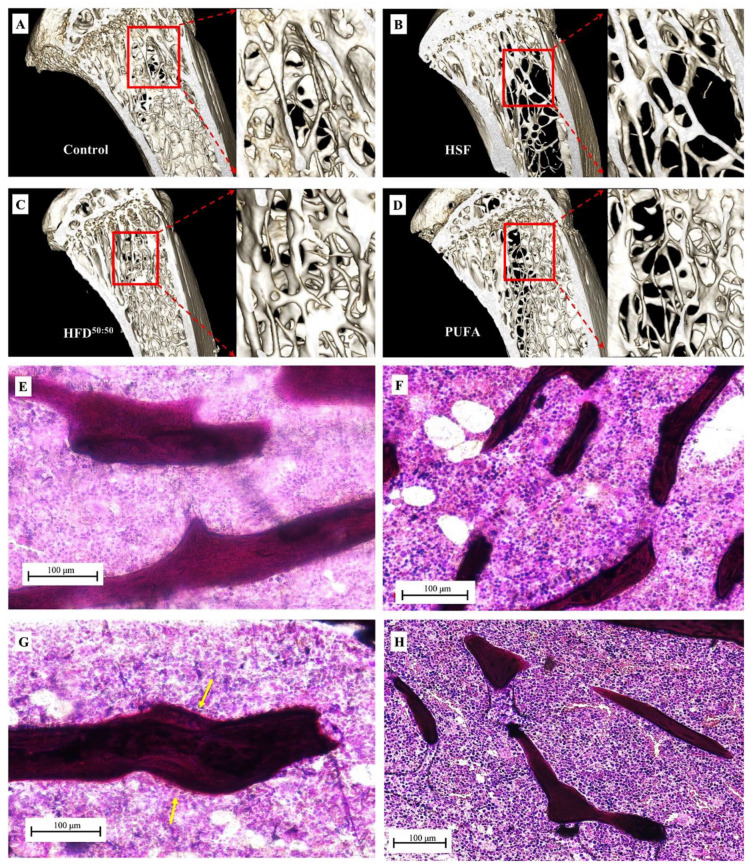
3D models of the proximal tibia were created using 3D Slicer. (**A**) Control, (**B**) HSF-fed group showing evidence of trabecular bone thinning and bone loss, (**C**) HFD^50:50^ diet demonstrating thicker and denser trabeculae when compared with all other groups and (**D**) the PUFA-fed group shows increased porosity compared with the control-fed and HFD^50:50^-fed animals. Micrographs taken using light transmission microscopy of cancellous bone in the proximal tibia in the (**E**) control, (**F**) HSF, (**G**) HFD^50:50^ and (**H**) PUFA groups. Increased trabecular thickness was observed in the HFD^50:50^ group, with areas of osteogenesis (yellow arrows) observed on the trabecular surface. In contrast, the surfaces of bone appeared quiescent in all other experimental groups, with thinner trabeculae seen in the HSF and PUFA samples.

When the trabecular and cortical features within the proximal tibiae were assessed, results showed increased trabecular length in the HFD^50:50^ group (458.1 ± 114.00 μm) and lowest in the HSF group (282.60 ± 64.87 μm). However, no statistically significant difference between groups was found (Figure 6A). The mean trabecular width was significantly increased in the HFD^50:50^-given animals (78.47 ± 8.17 μm) when compared with all other groups (PUFA (56.52 ± 6.81 μm) *p* = 0.031 (control (46.50 ± 2.70 μm) *p* = 0.002 and HSF (38.50 ± 2.13 μm), *p* = 0.001) (Figure 6B). Mean cortical thickness was significantly increased in HFD^50:50^-fed animals (153.40 ± 16.90 μm) and thinnest in the HSF group (92.50 ± 17.39 μm) (Figure 6C). Significantly increased thickness was measured in the HFD^50:50^ group when compared with the animals fed with an HSF diet (*p* = 0.042). The distance between trabeculae was significantly increased in the HSF group when compared with the control (*p* = 0.039) and HFD^50:50^ group (*p* = 0.018). No other differences were found (Figure 6D). Increased %bone area was measured in the HFD^50:50^ group and least in the HSF, with increase porosity measured in the PUFA and HSF groups. However, no significant differences were found (Figure 6E,F). Overall, increased trabecular bone was observed in the HFD^50:50^ group with the least in the HSF group. These results were supported by 3D reconstructed images of the tibia (Figure 7A–D) and qualitative histological assessment (Figure 7E–H). Results showed thicker trabeculae in sections prepared through tibia of mice fed a HFD^50:50^ diet, with evidence of ongoing osteogenesis. In contrast, the development of osteoporosis was observed in the HSF and PUFA groups, as shown by trabeculae that appeared thinner when compared with control- and HFD^50:50^-fed mice.

### 3.5. Tibial Morphometrics, BMD, BMC and BV/TV% 

Results for the structural morphometric parameters of control, HSF, HFD and PUFA tibiae are shown in Table 5. Results showed that tibial length was lowest in the HSF group and significantly decreased when compared with the HFD^50:50^ (*p* = 0.004)-fed animals. The longest tibiae were measured in the HFD^50:50^ group, however, no significant differences were found when compared with control and PUFA groups. The increase in the outer diameter was significantly greater in HSF animals when compared with both control (*p* = 0.047) and HFD^50:50^ (*p* = 0.009)-fed animals. When the moment of inertia was compared between groups, results showed a significant increase in the HSF group when compared to animals in the chow and HFD^50:50^ groups (*p* = 0.047 and *p* = 0.009, respectively). No other significant differences were found.

Qualitative analysis of μCT images (*n* = 1) of the trabecular architecture through the same region in each group, showed a loss of trabeculae and a more porous bone structure in each of the high-fat diet groups when compared with control-fed animals [Supplementary Figure S2A(a–d)]. However, no change in cortical bone was noted [Supplementary Figure S2A(e–h)]. Whole tibia BMD and BMC were highest in control-fed animals.

Levels of BMD were evaluated from the proximal (block 1) to distal (block 7) regions along the tibia [Supplementary Figure S2B and Supplementary Table S12]. Bone mineral content was demonstrated to increase from block 1–7 in the control-fed animals when compared with each of the high-fat diet groups [Supplementary Figure S2C and Supplementary Table S13]. The BV/TV fraction varied along the length of the tibia and results showed an increased BV/TV% in all 7 regions in the HFD^50:50^ group when compared with all other groups [Supplementary Figure S2D and Supplementary Table S14]. When BMD, BMC and BV/TV% were compared in the antero-posterior and medio-lateral planes, results showed increased BMD in the control-fed animals in all four aspects when compared with all animals in the high-fat diet groups [Supplementary Figure S2E and Supplementary Table S15]. In control-fed mice, BMC levels were increased anteriorly when compared with the lateral, medial and posterior regions of the tibiae (Supplementary Figure S2F). A similar trend was observed within animals in each of the high-fat diet groups (Supplementary Tables S16–S18). Animals in the control group demonstrated an increased BV/TV fraction in the anterior sector when compared with the medial and posterior sectors [Supplementary Figure S2]. This trend was observed in each of the high-fat diet groups.

## 4. Discussion

Fragility fractures are associated with significant morbidity, mortality and disability and are a growing major concern for public health globally [1,2,3]. The influence of diet on the development and progression of osteoporosis as well as on overall bone health, can be significant but is not fully understood. The aim of this study was to determine the effect of a high-fat diet of varying saturated FAs, MUFA and PUFA levels on the development of osteoporosis as indicated by alterations in bone area, architecture, mineral content, viscoelasticity, and resistance to fracture. The intake of ω-6 and ω-9 levels varied, with highest ω-6 in the PUFA diet and ω-9 in the HFD^50:50^-fed mice. Similar to other studies [6,12,13,14], our results showed that mice fed an HSF diet displayed increased cortical microcrack formation and propagation, a greater cross-sectional moment of inertia, tibiae that were shorter in length, and increased cancellous porosity, when compared with control-fed mice; key features characteristic of osteoporosis. Interestingly, and following adjustment to weight, overall bone strength in the HSF group was similar to control-fed mice. However, it is conceivable that the onset of osteoporosis would continue to increase over a longer time duration, resulting in further loss in mass and strength and a bone structure increasingly susceptible to fragility fracture. Therefore, these results in part, support our hypothesis.

High-resolution imaging has shown that bone ultimately fails through delamination of mineralized collagen fibrils [36] and perturbation of either the mineral or organic components could dramatically impact fracture resistance. There are two main intrinsic and extrinsic mechanisms that determine mechanical behavior. The intrinsic mechanism concerns plastic deformation at small scale lengths (i.e., microcrack initiation), whereas extrinsic mechanisms refer to structural features on larger micrometer length-scales, that are able to resist crack growth through various crack-tip shielding mechanisms (e.g., crack arrest by cement lines) [37]. Our findings suggest differences in both the intrinsic and extrinsic properties of bone when fed diets of varying fat composition. A key finding was that mice fed an HSF diet displayed a significantly increased number of microcracks that were shorter in length, when compared with the control-, PUFA- and HFD^50:50^-fed mice. This may be due to alterations in the level of proteins found within the tissue. Protein networks within bone are able to repeatedly dissipate large amounts of energy as well as store energy and exhibit large cohesion and toughness, potentially offering fracture resistance to bone [38,39]. Furthermore, noncollagenous proteins are enriched in cement lines, lamellar surfaces and interfibrillar spaces of mineralized collagen fibrils, and have the potential to also influence the extrinsic properties of bone. Thurner and colleagues [40] showed that OPN deficiency resulted in a 30% decrease in fracture toughness, that was independent of bone mass, structure and porosity, suggesting an important role for OPN in preventing crack propagation. Levels of these proteins were not measured in this study. However, following 10 [41] and 12 weeks [42] of a HSF diet in a rat model, Gautam et al. and Tencerova et al. demonstrated significantly reduced levels of serum OCN and OPN. This suggests that reduced protein levels in the HSF-fed animals may have contributed to the increase in microcracks measured.

Viscoelasticity depends on tissue composition, and fatigue crack formation and propagation is sensitive to viscoelastic behavior [43]. Despite the significant increase in microcrack initiation, and decrease in microcrack propagation measured in HSF versus control bone, our DMA results showed no change in the elastic (storage loss) or viscous (loss modulus) components in these two groups. Microdamage has also been associated with a decrease in modulus [44], and although not significant, our findings support this concept, as the lowest elastic modulus was measured in the HSF group. We cautiously speculate a decrease in toughness within the intrinsic properties of the HSF group, potentially due to changes in the protein levels, which allowed for crack initiation, whereas the maintenance or enhancement of unknown extrinsic bone properties were able to arrest crack propagation. This may have led to the observed formation of more but shorter microcracks. It is also important to note that the cross-sectional moment of inertia significantly increased in the HSF-fed mice when compared with control- and HFD^50:50^-fed mice. Due to its shape, mouse tibiae are subjected to mainly bending and compression. The structurally adaptive bony response observed in the HSF group, may be due to a compensatory mechanism that advantageously positions the material further from the neutral axis, through periosteal apposition (increased outer diameter) and endosteal resorption (increased inner diameter). This would increase resistance to stress and strain, distributing forces over a larger area, reducing the risk of fracture while promoting lightness for efficient movement. However, the slender bone would also exhibit greater susceptibility to microdamage accumulation, and this concept is supported by the increased microcrack formation that was measured in the HSF group [45,46,47].

In contrast, mice fed a diet high in PUFA (ω-6) displayed cortical bone with no observable microcrack formation when compared with control-fed mice. A recent study by Mak et al. [48] investigated rodents fed a PUFA diet and reported no change in OCN and OPN serum levels. No changes in calcium and OCN following 10 weeks of consumption has also been described [18], with significantly increased levels of OCN measured by 12.9 weeks [49], and 25.7 weeks [19] of feed. Notably, after 22 weeks of PUFA, total procollagen type 1 N-terminal propeptide and osteoblastic function significantly increased when compared to control-fed rats [50]. In our study, it is conceivable that the diet high in PUFA, enhanced protein formation and collagen function, which may have subsequently improved the intrinsic properties of bone, limiting microcrack formation. However, future studies are needed to investigate this further. DMA results showed a trend for an overall reduction in elastic modulus, storage modulus and loss tangent in the PUFA group, suggesting a less viscous bone matrix with reduced damping properties had developed. Interestingly, mice who consumed the PUFA diet also showed increased overall levels of bone strength when compared with all other groups. The increase in ultimate, fracture and yield strength observed is similar to other studies over comparable feed durations [17,18,19]. Although not significant, tibiae in the PUFA group also showed a higher cross-sectional moment of inertia, with the bone potentially adjusting its cross-sectional area to become more mechanically robust. This would be at the expense of ductility and toughness but with greater resistance to fatigue. In our study, this theory may also be supported by the development of few microcracks, and the higher yield stress observed in the PUFA group. Importantly, and despite the overall increase in levels of bone strength observed in PUFA-fed mice, our findings suggest that any beneficial effects measured may be temporary, as the onset of osteoporosis was also apparent. When the histological characteristics of early osteoporosis are combined with the trend of a lower elastic modulus and loss tangent, it is conceivable that over a longer study duration, the bone in this group may become increasingly susceptible to fragility fracture.

Remarkably, and despite gaining a similar amount of weight to mice in the HSF and PUFA groups, HFD^50:50^-fed mice presented with longer tibiae, limited microcrack formation, and significantly increased cortical and trabecular thickness when compared with control-fed animals. The HFD^50:50^-fed mice also displayed significantly higher ultimate stress values compared to control-fed mice. Our findings also show that mice fed HFD^50:50^ demonstrated increased viscous energy loss both at 0.05 Hz where there was more movement of the organic component, and at 10 Hz where there is less organic movement. This may suggest that a diet high in ω-9 promoted a more fluid component within bone that may have prevented microdamage and increased structural strength.

Several studies evaluating the ω-3/ω-6 PUFA ratio or their respective levels have indicated a positive role for ω-3 PUFA over ω-6 PUFA [23,51,52]. Omega-6 fatty acids are reported to inhibit osteoblast function [53], increase adipogenesis [54] and are typically pro-inflammatory, increasing the production of factors that enhance bone resorption including, IL-1ß, IL-6 and TNFα [55] in vitro, whereas ω-3 favors osteoblastogenesis via several pathways, including the attenuation of various pro-inflammatory cytokines [56], by increasing nitric oxide production [57] and by promoting osteoblastic differentiation via increased insulin-like growth factor and parathyroid hormone [58]. Similarly, ω-9 has been reported to promote osteoblast function [59] and attenuate inflammation [60]. The effects of PUFA on osteoclasts remain unclear, although it has been reported that ω-3 FA may lead to decreased osteoclast maturation [61], whereas ω-3, ω-6 and ω-9 monounsaturated fatty acids are reported to inhibit osteoclast formation [59,62,63]. In this study, qualitative histological analysis showed that after 8 weeks, mice fed a diet high in ω-6 and ω-9 showed disparate effects. While the mice fed a diet high in ω-6 displayed an overall increase in bone strength compared to all other groups, histological analysis and 3D reconstructed modeling showed thinner trabeculae and cortices with few areas of active bone formation on the trabecular surface. In contrast, mice fed a diet high in ω-9, displayed an increase in bone volume, with thicker trabeculae and cortices, and areas of active osteogenesis. In all experimental groups, qualitative analyses were similar, and revealed no substantial increase in osteoclastic activity, or presence of Howship’s lacunae. Finally, the tibiae were shorter in length in the HSF group when compared with all other groups and longest in the HFD^50:50^ group, suggesting a potential critical role of dietary fat in skeletal growth.

This study had several limitations. First, the degree of crystallinity and changes in crystal size, number and distribution alter the elastic, plastic and viscoelastic properties of bone, and this parameter was not measured in this study [64,65]. Second, BMD, BMC and BV/TV% were measured in one animal in each group, and further investigation is required to evaluate changes in bone mineral distribution and its association with diet, microdamage and fracture. Third, this study did not determine collagen content and quality. Alterations in crosslinking have been shown to impact the intrinsic toughness, through the accumulation of nonenzymatic glycation end-products and stiffening of the type I collagen network, which has been correlated to increased fracture risk [66,67]. This may have also contributed to the alterations in the viscoelastic properties observed. Fourth, the biochemical characteristics of treated mice including, routine clinical laboratory data (e.g., glucose, triglycerides, creatine), protein levels (e.g., OCN, OPN, cortisol, leptin), and the fatty acid composition of serum and bone extracts were not investigated in this study. Further investigation is warranted to elucidate how the various FAs impact bone metabolism, growth, and the mechanical properties of bone. Finally, a longer duration study is needed to determine the longitudinal effect of these diets on the progression of osteoporosis and susceptibility to fracture.

While ω-3, ω-6, and ω-9 FAs are all important dietary fats with a role in supporting bone health, it is important to also take into account the need for an overall optimal balance in intake when considering whole body health. For example, when in moderation, linoleic acid (ω-6) is associated with improved heart health. However, higher dietary intake of some ω-6 (e.g., linoleic acid with arachidonic acid and its metabolites), and ω-9 (e.g., oleic acid) FAs have been associated with an increased risk of heart disease and mortality [68,69,70]. A further limitation to the study is that the type of ω-3, ω-6, and ω-9 FAs ingested was unknown. It is possible that the main ω-9 FA in lard was oleic acid, however, levels of the ω-9 FA erucic acid, were undetermined. This is of significance as erucic acid has been reported to cause myocardial lipidosis, heart lesions, and hepatic steatosis in rats [71]. This study did not evaluate the response of the critical organs to the experimental diets, and this remains an important consideration for future studies. Finally, it is important to note that factors including gender, age, and genetic determinants can affect fatty acid metabolism and potentially the role of diet in bone health, and any existing diseases could further magnify this effect [72].

## 5. Conclusions

In conclusion, each of the high-fat diets induced similar levels of obesity and loading. However, the bone response to diet varied. Our findings showed the detrimental effect of an HSF diet on bone health while also revealing the significant but disparate effects that ω-6 and ω-9 enriched diets prompted in energy consumption, viscoelasticity, levels of microdamage, and cortical and trabecular thickness. Notably, and over the 8-week period, both the ω-6 and ω-9 diets produced similar increases in biomechanical bone strength. It is conceivable that the increased fracture, ultimate tensile, and yield stresses observed in the PUFA and HFD^50:50^ groups, may be due in part, to more substantial changes in the viscoelastic component of bone and future investigation in this area is warranted. However, caution is necessary. While mice fed the diet high in ω-6 developed bone with increased overall strength over this 8-week period, due to the reduced stiffness, loss tangent, compensatory adaption of the structure, and development of an osteoporotic bone architecture, it is uncertain whether the mechanical protection observed is temporary. Therefore, these results in part support our hypothesis. In contrast, the HFD^50:50^ diet, with its high levels of ω-9, may provide a superior level of protection to bone through beneficial levels of stiffness, fracture resistance, yield strength and ultimate strength, potentially facilitated though an increased viscous component of bone. Further studies will pursue the mechanisms by which these fatty acids mediate viscoelasticity, architecture, and bone strength in the context of a high-fat diet.

## Figures and Tables

**Table 1:** Experimental diets and the estimated contribution of essential ω-3 and ω-6 fatty acids within each diet group. The HFD^50:50^ diet was composed of 9.3% PUFA (soybean) and 91.7% of a monounsaturated and saturated mix. The HSF diet consisted of a 3.7% PUFA (soybean), 9.3% lard (saturated and unsaturated FA mix) and 87% saturated fat. The PUFA diet consisted of 3.7% PUFA (soybean), 45.8% lard, 45.9% Safflower oil and 3.6% saturated fat. In terms of the essential PUFA contributions, the control diet consisted of soybean oil (~13% ω-3, ~55% ω-6 and 18% ω-9), lard (0% ω-3, ~6–10% ω-6 and ~44–47% ω-9) and cocoa butter (0% ω-3, 2.8% ω-6, 33% ω-9). The HFD^50:50^ contained soybean oil and lard only, the HSF group soybean oil, lard and coconut oil (0% ω-3 and ω-6, ~6% ω-9) and the PUFA diet contained soybean oil, lard and safflower oil (0% ω-3, ~77% ω-6, 12.5% ω-9).

Diet Type	Diet #	Fat ratio: (Unsaturated to Saturated)	Total Fat (kcal%)	ω-3%	ω-6%	ω-9%
Control Diet	D07020902	1:1	10	2.8	31.6	30.7
High Polyunsaturated Fat Diet	D06062303	3.3:1	60	0.48	43.0	29.0
High Fat Diet^50:50^	D12492	1:1	60	1.25	14.15	44.2
High Saturated Fat Diet	D06062302	1:10	60	0.48	2.95	10.0

**Table 2:** Murine diet with 10 or 60 kcal% fat and modification to fat sources and fat level. * Vitamin mix (V1001): vitamin A acetate (500,000 IU/g), vitamin D3 (100,000 IU/g), Vitamin E acetate (500 IU/g), menadione sodium bisulfate (62.5% menadione), Biotin (1.0%), cyanocobalamin (0.1%), Folic acid (0.2 g), nicotinic acid (3 g), calcium pantothenate (1.6 g), pyridoxine-HCl (0.7 g), riboflavin (0.6 g), thiamine HCl (0.6 g) and sucrose (978.42 g).

	Control Diet		HFD^50:50^		HSF		PUFA	
	g	kcal	g	kcal	g	kcal	g	kcal
Protein (% by wt)	19.2	20	26.2	20	26.2	20	26.2	20
Carbohydrate (% by wt)	67.3	70	26.3	20	26.3	20	26.3	20
Fat (% by wt)	4.3	10	34.9	60	34.9	60	34.9	60
Total kcal		100		100		100		100
kcal/g	3.85		5.24		5.24		5.24	
Ingredients								
Protein Casein, 80 Mesh	200	800	200	800	200	800	200	800
L-Cystine	3	12	3	12	3	12	3	12
Carbohydrate								
Corn Starch	500	2000	0	0	0	0	0	0
Maltodexrin 10	100	400	125	500	125	500	125	500
Sucrose	100	400	68.8	275	68.8	275	68.8	275
Cellulose, BW200	50	0	50	0	50	0	50	0
Lipid								
Soybean Oil	10	90	25	225	10	90	10	90
Lard	5	45	245	2205	25	225	130	1170
Coconut-Oil, Hydrogenated	0	0	0	0	235	2115	0	0
Safflower Oil	0	0	0	0	0	0	130	1170
Cocoa Butter	30	270	0	0	0	0	0	0
Mineral Mix S10026								
Dicalcium phosphate	13	0	13	0	13	0	13	0
Calcium carbonate	5.5	0	5.5	0	5.5	0	5.5	0
Potassium citrate, 1 H_2_O	16.5	0	16.5	0	16.5	0	16.5	0
Vitamin Mix V10001 *	10	40	10	40	10	40	10	40
Choline Bitartrate	2	0	2	0	2	0	2	0
FD&C Yellow Dye #5	0	0	0	0	0.025	0	0	0
FD&C Red Dye #40	0	0	0	0	0	0	0.025	0
FD&C Blue Dye #1	0	0	0.05	0	0.025	0	0.05	0
Total	1055	4057	773.85	4057	773.85	4057	773.85	4057

**Table 3:** Data are expressed as mean ± SE. Differences in food, water, energy consumption, and weight in each of the experimental groups. (*n* = 5 per group).

Variables	Control	PUFA	HFD^50:50^	HSF
Starting body weight (g)	23.84 ± 0.28	26.9 ± 0.45	25.26 ± 0.54	25.42 ± 0.37
Final body weight (g)	27.02 ± 0.25	41.22 ± 1.42	39.66 ± 2.1	36.24 ± 0.59
Cumulative gain in body weight (g)	3.2 ± 0.09	14.32 ± 1.39	14.42 ± 1.7	10.84 ± 0.45
Mean cumulative water consumed (mL)	191.7 ± 18.8	240.4 ± 23.9	199.4 ± 8.0	185.4 ± 8.58
Mean starting food intake (g)	21.04 ± 0.75	30.78 ± 0.68	24.66 ± 0.93	20.86 ± 0.76
Mean final food intake (g)	19.76 ± 0.74	34.38 ± 2.87	23.46 ± 0.54	20.88 ± 0.86
Mean starting energy consumed (kcal)	81.04 ± 2.89	161.14 ± 3.56	129.2 ± 4.83	109.36 ± 3.98
Mean final energy consumed (kcal)	76.1 ± 2.84	180.08 ± 14.69	122.94 ± 2.85	109.44 ± 4.46
Mean cumulative food consumed (kcal)	618.96 ± 15.80	1390.24 ± 37.01	953.36 ± 17.70	900.04 ± 16.76

**Table 4:** The structural dependent mechanical properties of control, HSF, HFD and PUFA tibiae. (*n* = 5 per group). PYD: post yield displacement.

Variables	Control	PUFA	HFD^50:50^	HSF
Yield Force (N)	13.05 ± 2.72	11.67 ± 4.94	13.43 ± 4.69	11.62 ± 2.91
Ultimate Force (N)	17.22 ± 4.42	17.50 ± 4.49	18.91 ± 2.02	14.95 ± 1.07
Fracture Force (N)	14.79 ± 5.04	14.56 ± 5.91	15.54 ± 2.89	13.71 ± 1.39
Stiffness (N/mm)	74.84 ± 27.51	81.12 ± 28.29	75.63 ± 28.57	52.84 ± 19.36
PYD (mm)	0.50 ± 0.31	0.45 ± 0.18	0.37 ± 0.15	0.33 ± 0.09

**Table 5:** The structural morphometric parameters of control, HSF, HFD and PUFA tibiae. (*n* = 5 per group).

Variables	Control	HFD^50:50^	HSF	PUFA
Tibial morphology				
Length (mm)	20.4 ± 0.51	22.6 ± 0.51	17.6 ± 0.60	19.5 ± 1.76
Outer diameter (mm)	1.45 ± 0.11	1.41 ± 0.04	1.61 ± 0.08	1.45 ± 0.20
Inner diameter (mm)	0.89 ± 0.07	0.91 ± 0.03	1.07 ± 0.05	0.95 ± 0.16
Moment of inertia (m^4^)	1.97 ± 0.6 × 10^−13^	1.65 ± 0.11 × 10^−13^	(2.74 ± 0.52) × 10^−13^	(2.07 ± 1.44) × 10^−13^

## Data Availability

The data that support the findings of this study are available from the corresponding author, [MO], upon reasonable request.

## Supplementary Materials

The following are available online at https://www.mdpi.com/article/10.3390/nu14153165/s1, Table S1: Starting body weight (g) and the *p* values obtained when each of the groups were compared. * denotes a significant difference. Table S2: Final body weight (g) and the *p* values obtained when each of the groups were compared. * denotes a significant difference. Table S3: Total weight gain (g) over the 8-week study period and the *p* values obtained when each of the groups were compared. * denotes a significant difference. Table S4: Week 1 starting food intake (g) and the *p* values obtained when each of the groups were compared. * denotes a significant difference. Table S5: Week 8 final food intake (g) and the *p* values obtained when each of the groups were compared. * denotes a significant difference. Table S6: Cumulative food intake (kcal) over the 8-week study period and the *p* values obtained when each of the groups were compared. * denotes a significant difference. Table S7: The biomechanical strength properties at the tibial midpoint in each of the groups. Values are unadjusted according to body weight and tibial length. Table S8: [Unadjusted data]. A comparison of yield stress in each of the diet groups. * denotes a significant difference. Table S9: [Unadjusted data]. A comparison of ultimate stress in each of the diet groups. * denotes a significant difference. Table S10: Unadjusted data]. A comparison of fracture stress in each of the diet groups. * denotes a significant difference. Table S11: [Unadjusted data]. A comparison of elastic modulus in each of the diet groups. * denotes a significant difference. Table S12: Mean ± standard error BMD (g/cm^3^) of whole bone and by block. Table S13: Mean ± standard error BMC (g) of whole bone and by block. Table S14: Mean ± standard error BV/TV of whole bone and by block. Table S15: Tibial bone mineral and volume levels in the antero-posterior and medio-lateral aspects. Data obtained from 1 animal per group. Table S16: BMC in each of the four planes in mice in the HFD^50:50^ group. * denotes a significant difference. Table S17: BMC in each of the four planes in mice in the PUFA group. * denotes a significant difference. Table S18: BMC in each of the four planes in mice in the HSF group. * denotes a significant difference. Figure S1: [A–D] The mechanical properties of yield stress $\sigma_{y}$, ultimate stress $\sigma_{u}$, strength $\sigma_{f}$ and elastic modulus *E* at the tibial mid-point, varied between the different diet groups. The data have not been adjusted to body weight. Figure S2: [A(a–d)] Representative transverse μCT images showing the tibial cancellous bone region in the same location in the control, HSF, HFD^50:50^, and PUFA diet groups, respectively. Trabecular bone loss is noted in all high-fat diet groups when compared with the control-fed animals. [A(e–h)] representative transverse μCT images showing the tibial mid-cortical bone region in the same location in the control, HSF, HFD^50:50^, and PUFA diet groups, respectively. [B] BMD, [C] BMC and [D] BV/TV% from the proximal to distal region of the tibia. [E] BMD, [F] BMC and [G] BV/TV% in the AP/ML sectors. The BV/TV fraction measured in the HFD^50:50^ group, supports the histomorphometry result obtained (*n* = 1).
