# Supplementary material for: Omega-9 Modifies Viscoelasticity and Augments Bone Strength and Architecture in a High-Fat Diet-Fed Murine Model
Source: Nutrients. 2022 Jul 31;14(15):3165. doi: 10.3390/nu14153165 (PMC9370223; doi:10.3390/nu14153165)
Supplement: Supplementary file 1 [file nutrients-14-03165-s001.zip › nutrients-1832278-supplementary.pdf]

Supplementary Figures and Tables

Omega-9 Modifies Viscoelasticity and Augments Bone Strength and Architecture in a High-Fat Diet-Fed Murine Model

Mahmoud Omer*, Hessein Ali, Nina Orlovskaya, Amelia Ballesteros, Vee San Cheong, Kari Martyniak, Fei Wei, Boyce E. Collins, Sergey N. Yarmolenko, Jackson Asiatico, Michael Kinzel, Christopher Ngo, Jagannathan Sankar, Ashley Calder, Timothy Gilbertson, Teerin Meckmongkol, Ranajay Ghosh, Melanie Coathup

*Corresponding author

Table S1. Starting body weight (g) and the *p* values obtained when each of the groups were compared. *denotes a significant difference.

	Control	$HFD^{50:50}$	HSF	PUFA
Control				
$HFD^{50:50}$	0.056			
HSF	0.016*	0.834		
PUFA	0.009*	0.047*	0.047*	

Table S2: Final body weight (g) and the *p* values obtained when each of the groups were compared. *denotes a significant difference

	Control	$HFD^{50:50}$	HSF	PUFA
Control				
$HFD^{50:50}$	0.009*			
HSF	0.009*	0.209		
PUFA	0.009*	0.465	0.016*	

Table S3: Total weight gain (g) over the 8-week study period and the *p* values obtained when each of the groups were compared. *denotes a significant difference

	Control	$HFD^{50:50}$	HSF	PUFA
Control				
$HFD^{50:50}$	0.008*			
HSF	0.009*	0.075		
PUFA	0.008*	0.916	0.0028*	

Table S4: Week 1 starting food intake (g) and the *p* values obtained when each of the groups were compared. *denotes a significant difference

	Control	$HFD^{50:50}$	HSF	PUFA
Control				
$HFD^{50:50}$	0.016*			
HSF	0.917	0.016*		
PUFA	0.009*	0.009*	0.009*	

Table S5: Week 8 final food intake (g) and the *p* values obtained when each of the groups were compared. *denotes a significant difference

	Control	$HFD^{50:50}$	HSF	PUFA
Control				
$HFD^{50:50}$	0.021*			
HSF	0.248	0.076		
PUFA	0.009*	0.028*	0.016*	

Table S6: Cumulative food intake (kcal) over the 8-week study period and the p values obtained when each of the groups were compared. *denotes a significant difference

	Control	$HFD^{50:50}$	HSF	PUFA
Control				
$HFD^{50:50}$	0.009*			
HSF	0.009*	0.175		
PUFA	0.009*	0.009*	0.009*	

Table S7: The biomechanical strength properties at the tibial midpoint in each of the groups. Values are unadjusted according to body weight and tibial length

Variables	*Control*	*$HFD^{50:50}$*	*HSF*	*PUFA*
Biomechanical strength properties at tibial midpoint				
Yield stress σy (MPa)	77.32 ± 10.96	85.74 ± 10.94	52.08 ± 5.27	85.28 ± 13.69
Ultimate stress σu $(MPa$	104.68 ± 19.85	122.88 ± 6.51	67.57 ± 4.55	108.69 ± 15.34
Fracture stress σf (MPa)	91.68 ± 20.74	101.98 ± 11.51	62.42 ± 5.98	87.74 ± 15.43
Elastic modulus E (GPa)	2.54 ± 0.53	1.94 ± 0.18	1.03 ± 0.19	1.80 ± 0.38

Table S8: [Unadjusted data]. A comparison of yield stress in each of the diet groups. *denotes a significant difference

	Control	$HFD^{50:50}$	HSF	PUFA
Control				
$HFD^{50:50}$	0.602			
HSF	0.251	0.028*		
PUFA	0.806	1.000	0.142	

Table S9: [Unadjusted data]. A comparison of ultimate stress in each of the diet groups. *denotes a significant difference

	Control	$HFD^{50:50}$	HSF	PUFA
Control				
$HFD^{50:50}$	0.754			
HSF	0.175	0.009*		
PUFA	1.000	0.806	0.086	

Table S10: [Unadjusted data]. A comparison of fracture stress in each of the diet groups. *denotes a significant difference

	Control	$HFD^{50:50}$	HSF	PUFA
Control				
$HFD^{50:50}$	0.917			
HSF	0.602	0.016*		
PUFA	0.806	0.327	0.221	

Table S11: [Unadjusted data]. A comparison of elastic modulus in each of the diet groups. *denotes a significant difference

	Control	$HFD^{50:50}$	HSF	PUFA
Control				
$HFD^{50:50}$	0.251			
HSF	0.028*	0.009*		
PUFA	0.327	0.806	0.086	

Table S12: Mean ± standard error BMD (g/cm^3) of whole bone and by block.

Variable	*Control*	*$HFD^{50:50}$*	*HSF*	*PUFA*
Mean BMD (g/cm^3) Whole bone	0.53 ± 0.03	0.50 ± 0.02	0.48 ± 0.02	0.51 ± 0.03
Mean BMD by block				
1	0.32 ± 0.02	0.31 ± 0.02	0.30 ± 0.02	0.26 ± 0.01
2	0.43 ± 0.02	0.41 ± 0.02	0.39 ± 0.02	0.40 ± 0.02
3	0.47 ± 0.03	0.45 ± 0.03	0.43 ± 0.02	0.46 ± 0.02
4	0.51 ± 0.04	0.49 ± 0.04	0.49 ± 0.02	0.51 ± 0.03
5	0.56 ± 0.03	0.54 ± 0.03	0.52 ± 0.02	0.56 ± 0.03
6	0.65 ± 0.02	0.61 ± 0.02	0.58 ± 0.01	0.64 ± .008
7	0.73 ± 0.03	0.70 ± .007	0.67 ± .006	0.73 ± 0.06

Table S13: Mean ± standard error BMC (g) of whole bone and by block.

Variable	*Control*	*HFD$^{50:50}$*	*HSF*	*PUFA*
Mean BMC (g) Whole bone	0.38 ± 0.02	0.31 ± 0.01	0.32 ± 0.01	0.29 ± 0.01
Mean BMC by block				
1	0.45 ± 0.05	0.37 ± 0.03	0.38 ± 0.04	0.29 ± 0.02
2	0.40 ± 0.04	0.31 ± 0.03	0.33 ± 0.03	0.29 ± 0.02
3	0.37 ± 0.05	0.31 ± 0.04	0.31 ± 0.05	0.29 ± 0.05
4	0.35 ± 0.06	0.30 ± 0.05	0.31 ± 0.05	0.29 ± 0.05
5	0.35 ± 0.04	0.30 ± 0.04	0.30 ± 0.04	0.28 ± 0.03
6	0.36 ± 0.02	0.30 ± 0.02	0.29 ± 0.02	0.28 ± 0.02
7	0.35 ± 0.01	0.30 ± 0.008	0.30 ± 0.01	0.30 ± 0.01

Table S14: Mean ± standard error BV/TV of whole bone and by block.

Variable	*Control*	*HFD$^{50:50}$*	*HSF*	*PUFA*
Mean BV/TV Whole bone	0.59 ± 0.02	0.62 ± 0.02	0.56 ± 0.02	0.59 ± 0.02
Mean BV/TV by block				
1	0.43 ± 0.03	0.50 ± 0.03	0.42 ± 0.04	0.39 ± 0.03
2	0.53 ± 0.07	0.58 ± 0.07	0.50 ± 0.06	0.52 ± 0.06
3	0.57 ± 0.06	0.61 ± 0.04	0.53 ± 0.04	0.56 ± 0.04
4	0.61 ± 0.06	0.62 ± 0.05	0.59 ± 0.03	0.62 ± 0.05
5	0.62 ± 0.04	0.65 ± 0.04	0.61 ± 0.04	0.63 ± 0.05
6	0.66 ± 0.01	0.66 ± 0.02	0.61 ± 0.02	0.65 ± 0.02
7	0.72 ± 0.03	0.72 ± 0.01	0.67 ± 0.01	0.74 ± 0.05

Table S15: Tibial bone mineral and volume levels in the antero-posterior and medio-lateral aspects. Data obtained from 1 animal per group.

Variables	*Control*	*HFD$^{50:50}$*	*HSF*	*PUFA*
AP-ML tibia bone mineral and volume levels (sum)				
BMD (mg.cm^{-2}):				
Anterior	3.79	3.70	3.57	3.55
Lateral	3.98	3.82	3.52	3.81
Medio	3.38	3.23	3.19	3.33
Posterior	3.55	3.31	3.26	3.54
BMC (mg):				
Anterior	3.09	2.59	2.63	2.31
Lateral	2.7	2.22	2.24	2.04
Medio	1.87	1.59	1.63	1.54
Posterior	2.88	2.33	2.38	2.21
BV/TV%:				
Anterior	4.55	4.81	4.39	4.41
Lateral	4.35	4.46	3.98	4.27
Medio	3.85	4.11	3.75	3.93
Posterior	3.80	3.97	3.63	3.84

Table S16: BMC in each of the four planes in mice in the HFD$^{50:50}$ group. *denotes a significant difference

	Lateral	Anterior	Medial	Posterior
Lateral				
Anterior	0.048*			
Medial	0.003*	0.002*		
Posterior	0.338	0.142	0.003*	

Table S17: BMC in each of the four planes in mice in the PUFA group. *denotes a significant difference

	Lateral	Anterior	Medial	Posterior
Lateral				
Anterior	0.035*			
Medial	0.018*	0.006*		
Posterior	0.035*	0.338	0.009*	

Table S18: BMC in each of the four planes in mice in the HSF group. *denotes a significant difference

	Lateral	Anterior	Medial	Posterior
Lateral				
Anterior	0.085			
Medial	0.004*	0.003*		
Posterior	0.482	0.142	0.004*	

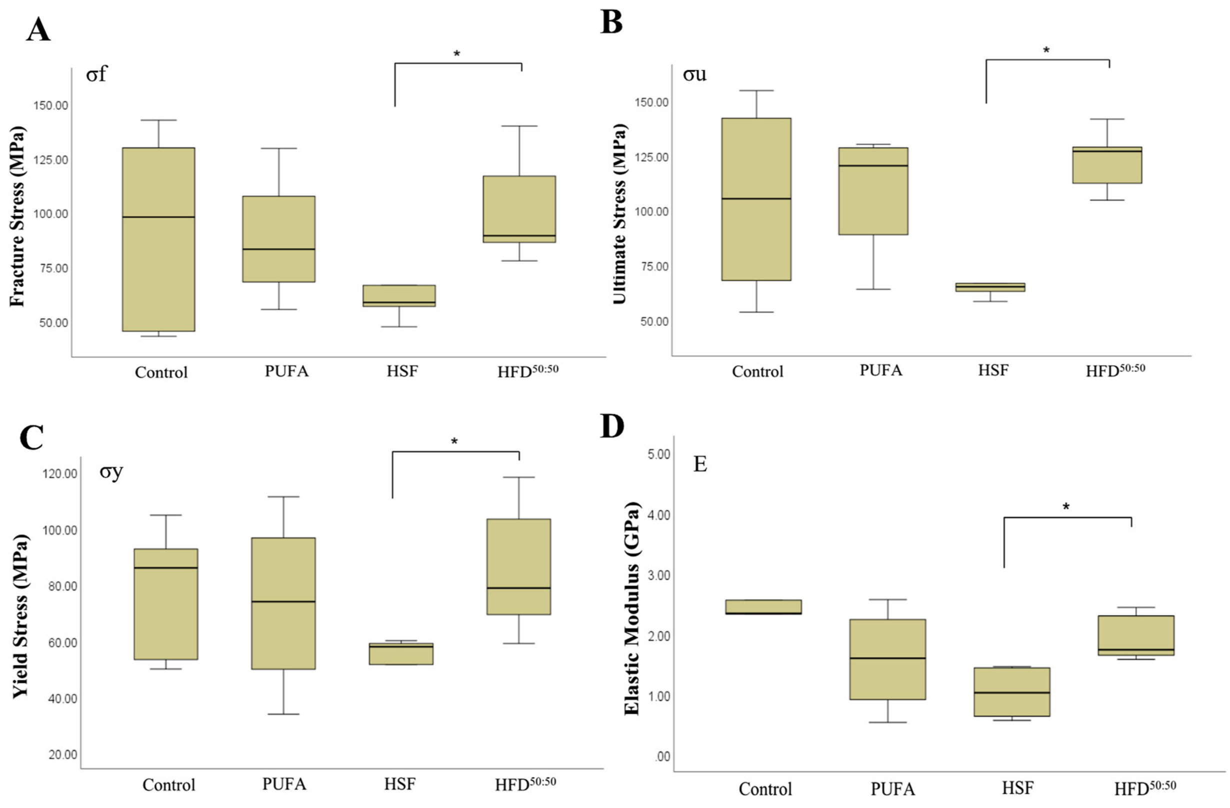

Figure S1. [A-D] The mechanical properties of yield stress σ_y, ultimate stress σ_u, strength σ_f and elastic modulus E at the tibial mid-point, varied between the different diet groups. The data has not been adjusted to body weight

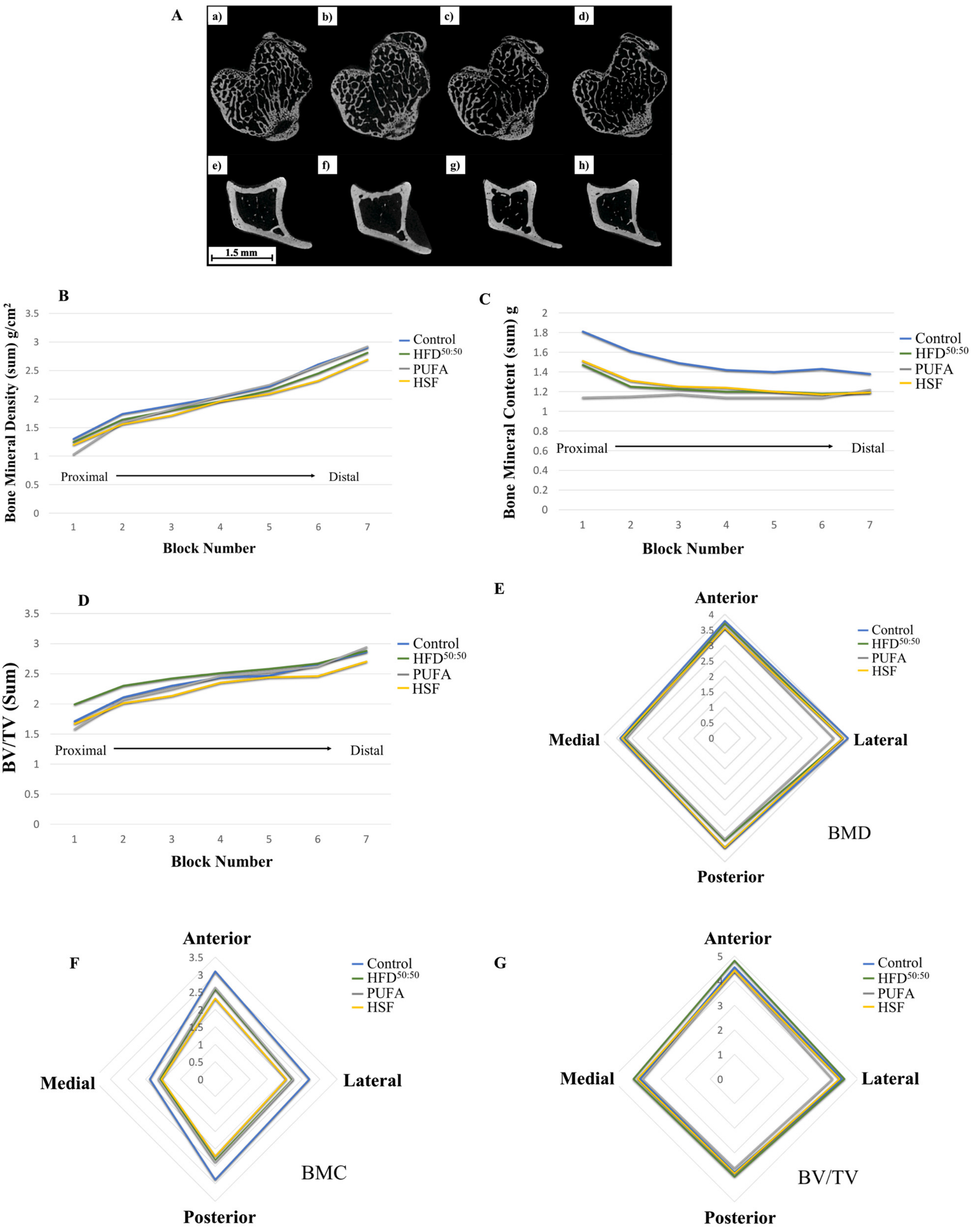

Figure S2. [A(a, b, c, d)] Representative transverse μCT images showing the tibial cancellous bone region in the same location in the control, HSF, HFD$^{50:50}$, and PUFA diet groups respectively. Trabecular bone loss is noted in all high-fat diet groups when compared with the control fed animal. [A(e, f, g, h)] representative transverse μCT images showing the tibial mid-cortical bone region in the same location in the control, HSF, HFD$^{50:50}$, and PUFA diet groups respectively. [B] BMD, [C] BMC and [D] BV/TV% from the proximal to distal region of the tibia. [E] BMD, [F] BMC and [G]

BV/TV% in the AP/ML sectors. The BV/TV fraction measured in the HFD$^{50:50}$ group, supports the histomorphometry result obtained (*n*=1).

No significant differences in BMD were found. However, BMC was significantly higher in the chow fed animal when compared with the HFD$^{50:50}$, HSF and PUFA groups (p = 0.002, 0.007 and <0.0001 respectively). The BV/TV% fraction was highest in the HFD$^{50:50}$ group and significantly increased when compared with the HSF group only (p = 0.017). When levels of BMD were evaluated from the proximal (block 1) to distal (block 7) regions along the tibia [**Supplementary Figure S2** B and **Supplementary Table S12**], results showed lowest values in the HSF group, with a significantly less BMD found in block 6 when compared with the control (p = 0.043) and PUFA (p = 0.021) groups. In block 7, significantly lower BMD was measured in the HSF-fed animal when compared with those who received a HFD$^{50:50}$ diet (p = 0.021). No other significant differences were found. Bone mineral content was demonstrated to increase from block 1 – 7 in the control fed animal when compared to each of the high-fat diet groups. A significantly lower BMC was measured in the PUFA group when compared with the control-fed mouse in block 1 (p = 0.043) [**Supplementary Figure S2** C and **Supplementary Table S13**]. In block 7, BMC was significantly lower in all high-fat diet groups when compared with the control fed animal (HFD$^{50:50}$, p = 0.021; HSF, p = 0.043; PUFA, p = 0.043). No other significant differences were found. The BV/TV fraction varied along the length of the tibia and results showed an increased BV/TV% in all 7 regions in the HFD$^{50:50}$ group when compared to all other groups [**Supplementary Figure S2** D and **Supplementary Table S14**]. Significantly increased values were demonstrated in block 7 and when compared with the HSF group (p = 0.021). No other significant differences were found. When BMD, BMC and BV/TV% were compared in the antero-posterior and medio-lateral planes, results showed increased BMD in the control fed animals in all four aspects when compared with all animals in the high-fat diet groups, however no significant differences were found. In all groups, BMD was highest in the lateral aspect and lowest medially [**Supplementary Figure S2** E and **Supplementary Table S15**]. In control fed mice, BMC levels were significantly increased anteriorly when compared with the lateral, medial and posterior regions of the tibiae (p = 0.002 in all cases; **Supplementary Figure S2** F). A similar trend was observed within animals in each of the high-fat diet groups (statistical comparisons are presented in **Supplementary Tables S16-S18**). Significantly increased BMC was measured in the control fed mice when compared in the lateral (HFD$^{50:50}$, p = 0.013; HSF, p = 0.018; PUFA p = 0.002) and anterior (HFD$^{50:50}$, p = 0.025; HSF, p = 0.035; PUFA p = 0.035) aspects when compared with the high-fat fed animals. Posteriorly, significantly increased BMC was measured in the control group when compared with PUFA fed animals (p = 0.002). No other significant differences were found. Animals in the control group demonstrated a significantly increased BV/TV fraction in the anterior sector when compared with the medial (p = 0.025) and posterior (p = 0.048) sectors [**Supplementary Figure S2** G]. This trend was observed in each of the high-fat diet groups however, no significant differences were found.
